# Investigating Intervention Components and Exploring States of Receptivity for a Smartphone App to Promote Physical Activity: Protocol of a Microrandomized Trial

**DOI:** 10.2196/11540

**Published:** 2019-01-31

**Authors:** Jan-Niklas Kramer, Florian Künzler, Varun Mishra, Bastien Presset, David Kotz, Shawna Smith, Urte Scholz, Tobias Kowatsch

**Affiliations:** 1 Center for Digital Health Interventions Institute of Technology Management University of St. Gallen St. Gallen Switzerland; 2 Center for Digital Health Interventions Department of Management, Technology and Economics Swiss Federal Institute of Technology Zurich Switzerland; 3 Department of Computer Science Dartmouth College Hanover, NH United States; 4 Institute of Sports Studies University of Lausanne Lausanne Switzerland; 5 Center for Technology and Behavioral Health Geisel School of Medicine at Dartmouth Lebanon, NH United States; 6 Institute for Social Research University of Michigan - Ann Arbor Ann Arbor, MI United States; 7 Medical School University of Michigan - Ann Arbor Ann Arbor, MI United States; 8 Department of Psychology University of Zurich Zurich Switzerland

**Keywords:** physical activity, mHealth, walking, smartphone, incentives, self-regulation

## Abstract

**Background:**

Smartphones enable the implementation of just-in-time adaptive interventions (JITAIs) that tailor the delivery of health interventions over time to user- and time-varying context characteristics. Ideally, JITAIs include effective intervention components, and delivery tailoring is based on effective moderators of intervention effects. Using machine learning techniques to infer each user’s context from smartphone sensor data is a promising approach to further enhance tailoring.

**Objective:**

The primary objective of this study is to quantify main effects, interactions, and moderators of 3 intervention components of a smartphone-based intervention for physical activity. The secondary objective is the exploration of participants’ states of receptivity, that is, situations in which participants are more likely to react to intervention notifications through collection of smartphone sensor data.

**Methods:**

In 2017, we developed the *A*ssistant to *L*ift your *L*evel of activit*Y* (Ally), a chatbot-based mobile health intervention for increasing physical activity that utilizes incentives, planning, and self-monitoring prompts to help participants meet personalized step goals. We used a microrandomized trial design to meet the study objectives. Insurees of a large Swiss insurance company were invited to use the Ally app over a 12-day baseline and a 6-week intervention period. Upon enrollment, participants were randomly allocated to either a financial incentive, a charity incentive, or a no incentive condition. Over the course of the intervention period, participants were repeatedly randomized on a daily basis to either receive prompts that support self-monitoring or not and on a weekly basis to receive 1 of 2 planning interventions or no planning. Participants completed a Web-based questionnaire at baseline and postintervention follow-up.

**Results:**

Data collection was completed in January 2018. In total, 274 insurees (mean age 41.73 years; 57.7% [158/274] female) enrolled in the study and installed the Ally app on their smartphones. Main reasons for declining participation were having an incompatible smartphone (37/191, 19.4%) and collection of sensor data (35/191, 18.3%). Step data are available for 227 (82.8%, 227/274) participants, and smartphone sensor data are available for 247 (90.1%, 247/274) participants.

**Conclusions:**

This study describes the evidence-based development of a JITAI for increasing physical activity. If components prove to be efficacious, they will be included in a revised version of the app that offers scalable promotion of physical activity at low cost.

**Trial Registration:**

ClinicalTrials.gov NCT03384550; https://clinicaltrials.gov/ct2/show/NCT03384550 (Archived by WebCite at http://www.webcitation.org/74IgCiK3d)

**International Registered Report Identifier (IRRID):**

DERR1-10.2196/11540

## Introduction

### Background

Mobile health (mHealth) and sensing technologies recently sparked excitement because of their capability to deliver large-scale personalized behavior change interventions at low cost [1], which can potentially reduce the disease burden associated with health behaviors, such as diet behavior, smoking, or physical inactivity [2]. Beyond passive monitoring of health behavior, smartphones and wearables collect a wealth of contextual information (such as time, location, communication logs, or physical activities) that can be used to tailor the delivery of interventions to participant states that increase the intervention’s likelihood of success. For example, an intervention could only be delivered at points in time when the participant is likely to change her or his behavior (state of opportunity) or is likely to engage with the intervention content (state of receptivity) [3]. mHealth apps that utilize this kind of dynamic tailoring are referred to as just-in-time adaptive interventions (JITAIs) [3].

During the development process of a JITAI, it is crucial to decide what key intervention components are needed to affect the desired intervention outcome and what information should be used to tailor the delivery of each component to participants over time [4]. The first question involves an empirical evaluation of single candidate intervention components. The second question involves identifying effective time-varying moderators that indicate in which situations the intervention component is or is not effective. Unfortunately, these decisions can hardly be informed by past research because traditional study designs (eg, randomized controlled trials) rarely evaluate single intervention components or time-varying moderators of intervention effects. To facilitate the development of JITAIs, Klasnja et al, therefore, proposed the microrandomized trial (MRT) [5].

The goals of an MRT are to quantify proximal (short-term) main effects of single intervention components, to investigate how these effects change over time, and to identify which contextual variables moderate the effects of single intervention components. MRTs use repeated randomization of participants to different versions, or presence and absence, of individual intervention components over time, which enables estimation of time-averaged main effects of single intervention components on proximal outcomes as well as time-varying effects and their contextual moderators. Results of an MRT can consequently inform the researcher which components to include in an optimized version of the intervention and how to adapt the delivery of each intervention component to maximize effectiveness.

Although MRTs are designed to accommodate contextual moderation, context is likely to be multidimensional —for example, not just time or location but rather the nexus of time and location (or other higher order interactions) define opportune moments for intervention. This limits the approach of investigating single variables as potential tailoring variables within MRTs. A potential way of overcoming this limitation is to train machine learning models that classify the participants’ latent *states* of intervention receptivity or vulnerability given a vector of high-resolution smartphone sensor data. Research on interruptibility, for example, demonstrated that models trained on smartphone sensor data successfully predict the quality and quantity of participants’ reactions to notifications on their smartphone [6-8]. Thus, this approach could allow to continuously model each participant’s state of receptivity (ie, the likelihood of engaging with an intervention) from a vast number of variables. Predictions of these models can in turn be used to inform intervention delivery of a JITAI.

In this paper, we describe the rationale and design of a 6-week MRT that evaluates main effects and moderators of 3 different intervention components (self-monitoring prompts, planning, and incentives) of the *A*ssistant to *L*ift your *L*evel of activit*Y* (Ally), a smartphone app to promote physical activity. Ally delivers interventions via an interactive text-based chatbot interface and simultaneously collects contextual data using the smartphone’s built-in sensors. We also report descriptive statistics from our remote recruiting process and baseline characteristics of participants.

### Objectives

To inform the evidence-based development of JITAI for physical activity, the described study has the following objectives:

- To quantify main effects and interactions of main effects of 3 intervention components of Ally, an mHealth intervention for physical activity.
- To explore how the effects of intervention components are moderated by contextual factors and change over time.
- To collect a wide range of high-resolution smartphone sensor data to predict the participants’ states of receptivity.

## Methods

### Study Setting

This study is part of a research collaboration between the Center for Digital Health Interventions, a joint initiative of the Department of Management, Technology, and Economics at ETH Zurich and the Institute of Technology Management at the University of St. Gallen and the CSS insurance, a large health insurer in Switzerland. Data for this study were collected from October to December 2017 in the German-speaking part of Switzerland. The study is registered on ClinicalTrials.gov (NCT03384550) and was approved by the ethical committee of ETH Zurich.

### The Assistant to Lift Your Level of Activity App

**Figure 1:**
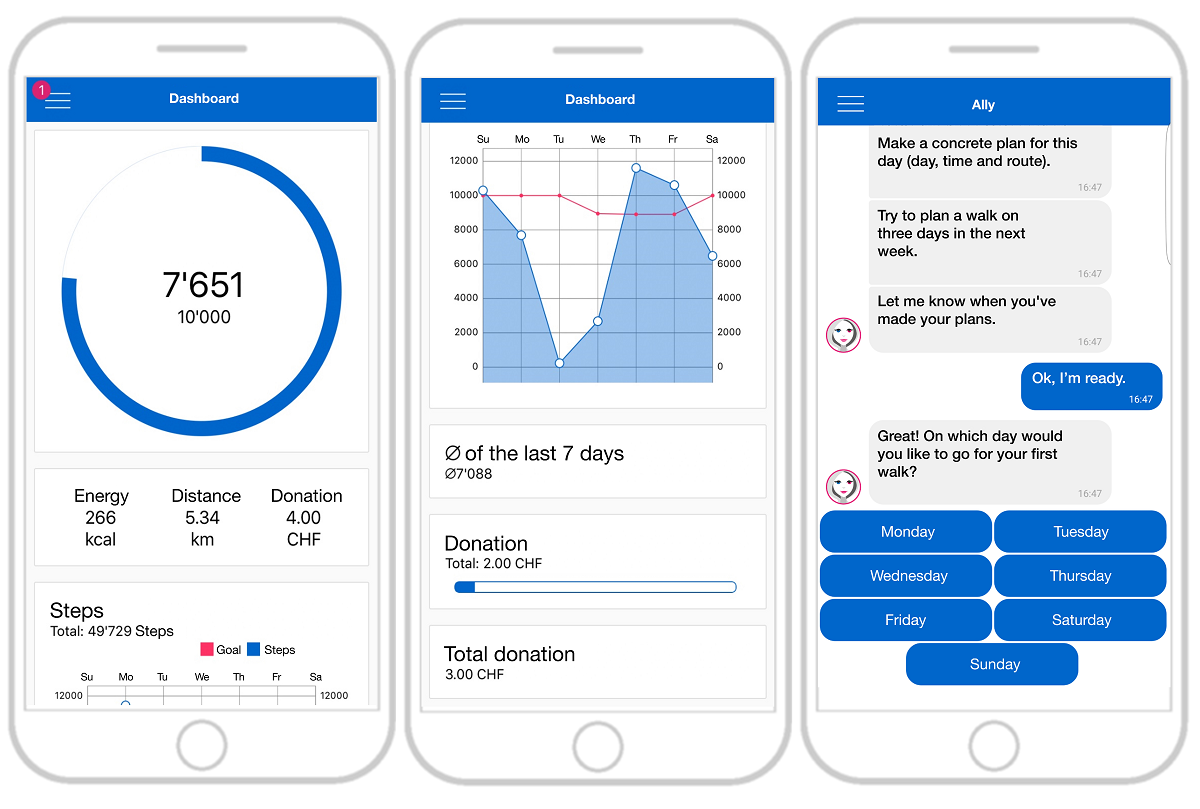
The Ally app: Dashboard with daily (left) and weekly overview (middle) and chat interactions with the Ally chatbot (right).

The Ally app focuses on measuring and increasing walking, a popular and safe activity [9,10] that is known to have positive health effects independent of other types of physical activity [11]. Steps per day as an objective measure of walking can be obtained from the majority of commercially available smartphones with acceptable accuracy [12]. The Ally smartphone app tracks participants’ daily step counts and provides interventions to help participants reach daily step goals. It contains a dashboard that displays basic information such as the participants’ current step count and the step goal of the current day as well as an activity overview of the past 7 days (Figure 1). Ally runs on the common operating systems Android and iPhone operating system (iOS). On Android smartphones, Ally obtains all physical activity–related information from GoogleFit, a health-tracking platform developed by Google. On iOS smartphones, the same information is obtained from the HealthKit, an application programming interface for health apps provided by Apple. To obtain smartphone sensor data, we used EmotionSense, a framework to support smartphone-based data collection originally developed for experimental social psychology research [13].

Step goals are personalized and calculated daily for each participant based on the participant’s activity over the past 9 days employing the moving-window percentile-rank algorithm described by Adams et al [14]. This adaptive goal-setting algorithm sets the daily step goal to the sixtieth percentile of the participant’s step count distribution of the past 9 days, meaning that the participant reaches her or his step goal 40% of the times when maintaining her or his recent activity level. Previous studies demonstrated that this adaptive goal setting outperforms static step goals [14,15]. To facilitate maintenance of behavior change, adaptive step goals are capped at 10,000 steps per day, which approximates the World Health Organization recommendations for physical activity [16,17].

To administer the intervention components evaluated in this study, the Ally app includes a chatbot (Ally) that provides interactive coaching dialogues similar to other messaging apps such as Apple’s iMessage, Facebook’s Messenger, or WhatsApp. The open source behavioral intervention platform MobileCoach [18] was used to build the chatbot and deliver the interactive coaching dialogues. In previous studies, MobileCoach-based interventions have successfully reduced problem drinking in adolescents [19] and engaged the majority of participants of a 3-month smoking cessation program [20]. Participants interact with Ally by selecting predefined answer options (Figure 1) that trigger a response by the chatbot according to the conversational rules specified in the MobileCoach system.

Beyond specific interventions, the chatbot also communicates the daily step goal in the morning and feedback regarding the goal together with informative facts about physical activity at 8 pm in the evening to all participants.

### Study Design

From October to December 2017, insurees of a large Swiss health insurance used the Ally app over a 12-day baseline and a 6-week intervention period. During the baseline period, participants only had access to the dashboard of the app, and no interventions were administered. Over the course of the 6-week intervention period, we randomized participants to different versions of 3 intervention components: daily self-monitoring prompts (2 levels; within-subjects), a weekly planning intervention (3 levels; within-subjects), and daily incentives (3 levels; between-subjects). The rationale for these intervention components is described below. To meet study objective three, we aimed to explore if and how participants’ reaction to intervention components were dependent on their context. To do so, we ideally need to observe reactions to intervention notifications in a wide variety of contexts. We, therefore, sent out intervention and step goal–related notifications at random points in time but within prespecified time windows that guaranteed delivery at appropriate times. For example, daily step goal notifications were delivered at a random point in time between 8 am and 10 am as users likely expect to be notified about their goal early in the day. Participants completed a Web-based questionnaire at baseline and at postintervention follow-up and received CHF 10 (US $10 as of 2017) for the successful completion of both questionnaires. If participants provided consent, they were invited to participate in exit interviews after the end of the study that investigate perceptions of participants and mechanisms of behavior change.

The following subsections first describe details and rationale for each intervention component as well as for potential moderators. Subsequently, we outline how each component was randomized during the intervention period and how we define the proximal outcome to evaluate each component.

### Intervention Components

#### Self-Monitoring Prompts

Self-regulatory processes have been identified as a key factor for health behavior change [21,22]. To support participants’ self-regulation, we designed short dialogue-based self- monitoring prompts. Self-monitoring prompts remind the participants of their daily step goal, compare the participants’ current step count to their daily goal, and provide an estimate of walking minutes necessary to reach the goal together with an actionable tip on how to increase physical activity. These dialogues were designed to support the 3 subprocesses of the self-regulatory construct action control, namely self-monitoring, awareness of goals or standards, and self-regulatory effort [23,24]. If a participant had already reached their daily step goal when starting the dialogue, she/he would receive positive and encouraging feedback from the Ally chatbot instead.

Participants were randomized to receive a self-monitoring prompt or no prompt every day during the intervention period except Sunday, as this day was reserved for the planning intervention (see below). Self-monitoring prompts were delivered at a random point in time between 10 am and 6 pm.

Participants’ general tendency to self-monitor their physical activity may affect the effect of self-monitoring prompts because the information provided by the prompt is likely to be redundant to participants who are already aware of their activity level. In addition, timing of the self-monitoring prompt may be critical. Research from cognitive psychology demonstrates that people assign more value to performance increases when their current performance is close to their goal [25]. Consequently, self-monitoring prompts may be more effective if they are sent at times when participants are closer to reaching their step goal.

#### Planning

Even if motivation to change exists, previous studies show that on average, 47% of people fail to act upon their good intentions [26]. Forming specific plans about when and how to act increases the likelihood of performing the intended behavior [27,28] and helps to bridge the so-called intention behavior gap. Planning can be further divided into action planning (AP; specifying when, where, and how to act) and coping planning (CP; specifying coping responses for barriers and difficult situations) [29]. Plans that are articulated in an if-then format (eg, “if I am tired at work, I will go for a brief walk to get new energy”) are typically referred to as implementation intentions [30].

Every Sunday during the intervention period, participants received either an AP, a CP, or no planning intervention (control; CC). In the AP condition, Ally asks the participant to plan at least one and up to 3 walks for the upcoming week. To plan a single walk, the participants need to specify the day of the week, the time, and the route that they intend to walk. To create flexible plans and thus increase the likelihood of adherence, Ally advises the participant to choose event-related times (eg, after work) instead of actual times. In the CP condition, Ally asks the participant to identify barriers for physical activity by reflecting on the 2 least active days from the previous week. The participant is then prompted to develop counterstrategies for each barrier using the if-then format of implementation intentions [30]. Ally guides this process using examples for common barriers for physical activity that have been identified in previous studies [31-33], for example: “If I want to go for a walk but I lack motivation, I will think of the benefits of walking for health to motivate myself.” Finally, the participant has the option to anticipate days of the upcoming week where the barrier may arise again. Both AP and CP include reminders for the participant on days when either a walk or a coping reaction was scheduled. To address the third objective of this study, planning interventions were sent out on Sundays at a random point in time between 10 am and 8 pm.

Participants’ activity level and contexts may moderate the effects of AP and CP. Participants with low activity levels may be more likely to benefit from AP, which promotes the initiation of action, whereas participants with high activity levels may benefit more from CP, which prevents routines from distraction [29,34]. Furthermore, completing the planning intervention can take several minutes and requires a considerable amount of the participants’ attention and cognitive capacity. Ideally, the planning intervention should, therefore, not be delivered in situations where the participant is involved in an attention-consuming activity, such as social activities or work.

#### Incentives

Meta-analyses [35,36] and recent randomized trials [37-39] have demonstrated the ability of financial incentives to increase physical activity. However, financial incentives may reduce intrinsic motivation [40,41]; thus, charity incentives have been proposed as an alternative incentive strategy. Charity incentives, that is, donations to a charity organization, could foster experiences of autonomy and relatedness, which are known to facilitate rather than impede the buildup of intrinsic motivation [42]. Moreover, 2 recent studies have so far compared financial and charity incentives with mixed results [37,43].

In this study, participants were randomly allocated to either a financial incentive, a charity incentive, or a control condition using an allocation ratio of 1:1:1. In the financial incentive condition, participants received CHF 1 (US $1 as of 2017) for each day they met their personalized step goal. In the charity incentive condition, instead of keeping the reward to themselves, participants made a donation of CHF 1 to a charity of their choice. Participants allocated to the control condition received no incentives. Earned rewards (maximum of CHF 42) were paid to participants or donated to charity after completion of the study.

We hypothesize that the presence of incentives moderates the effect of the other intervention components. Both planning and self-monitoring prompts target the participants’ self-regulatory processes and thus require the participant to be motivated to reach the provided step goals to produce an effect [44]. As we expect the incentives to increase the motivation of participants, we hypothesize that effects of self-monitoring prompts and planning are more pronounced for participants receiving financial or charity incentives.

### Randomization, Allocation Concealment, and Blinding

The MobileCoach version used in this study requires the time point of dissemination for all dialogues to be known a priori. Therefore, randomization had to be performed upon enrollment of participants for all intervention components. Each participant was randomized to 1 out of 3 incentive conditions using simple randomization and an allocation ratio of 1:1:1. In addition, participants were randomized to 1 out of 9 planning intervention sequences (S_1_-S_9_) that determine the order in which the participant received the AP intervention, the CP intervention, or CC intervention throughout the intervention period. We used blocked randomization with a block size of 9 to achieve balance between the sequences. The resulting intervention schedule (Table 1) is uniform and strongly balanced, which controls for time and carry-over effects [45]. To avoid interference of self-monitoring prompts and planning, self-monitoring prompts were not delivered on Sundays. Thus, subtracting 6 from 42 left 36 available days for delivering self-monitoring prompts. To prevent repetition of content, we created 18 different versions of self-monitoring prompts that we randomly allocated to the 36 days for each participant. Consequently, at each of the 36 days, half of participants received a self-monitoring prompt (on average), whereas the other half received no prompt. All randomizations were performed using random number sequences generated with the shuffle-array package in JavaScript.

The fully automated randomization process guarantees allocation concealment for everyone involved in the study. Variables in the dataset indicating intervention allocation are encrypted to blind members of the research team involved in data analysis. A researcher at the Swiss Federal Institute of Technology in Zurich who is not involved in data analyses holds the decryption key and is instructed to safely store the key until the analysis script has been finalized. Due to the setting of the study, it is not possible to blind participants to intervention assignments. To reduce the impact of performance and attrition bias, participants were not informed about the details of the intervention components before the study.

### Measurements

#### Primary and Secondary Outcomes

As the intervention components (see Table 2) are randomized on different timescales, we need to define primary and secondary proximal outcomes that correspond to these timescales to correctly evaluate the intervention components. The proportion of overall participant days that step goals are achieved during the intervention period is the primary outcome to evaluate the different incentive conditions. Weekly and daily proportions of participant days that step goals are achieved during the intervention period are the primary outcomes of the planning and self-monitoring prompts, respectively. On the same timescales, differences in steps per day measured with the smartphone are investigated as a secondary outcome.

**Table 1:** Intervention schedule of the planning intervention.

Sequence	Week 1	Week 2	Week 3	Week 4	Week 5	Week 6
S_1_	AP^a^	AP	CP^b^	CC^c^	CC	CP
S_2_	CP	CP	CC	AP	AP	CC
S_3_	CC	CC	AP	CP	CP	AP
S_4_	AP	CP	CP	AP	CC	CC
S_5_	CP	CC	CC	CP	AP	AP
S_6_	CC	AP	AP	CC	CP	CP
S_7_	AP	CC	CP	CP	CC	AP
S_8_	CP	AP	CC	CC	AP	CP
S_9_	CC	CP	AP	AP	CP	CC

^a^AP: action planning.

^b^CP: coping planning.

^c^CC: control condition (no planning).

**Table 2:** Overview of intervention components of the *A* ssistant to *L* ift your *L* evel of activit*Y* (Ally) app.

Component and intervention options		Randomization	Mode of delivery	Time of delivery	Behavior change techniques [48]^a^	Proximal outcome
**Self-monitoring prompts**						
	Prompt	Upon enrollment; allocation ratio 1:1	Chat	Daily except Sunday; randomly between 10 am and 6 pm	1.6; 2.2; 4.1	Daily proportion of participant days that step goals were achieved
	Control (no prompt)	Upon enrollment; allocation ratio 1:1	N/A^b^	N/A	N/A	Daily proportion of participant days that step goals were achieved
**Planning**						
	Action planning	Upon enrollment; allocation ratio 1:1:1	Chat	Sundays; randomly between 10 am and 6 pm	1.4	Weekly proportion of participant days that step goals were achieved
	Coping planning	Upon enrollment; allocation ratio 1:1:1	Chat	Sundays; randomly between 10 am and 6 pm	1.2	Weekly proportion of participant days that step goals were achieved
	Control (no planning)	Upon enrollment; allocation ratio 1:1:1	N/A	N/A	N/A	Weekly proportion of participant days that step goals were achieved
**Incentives**						
	Cash incentives	Upon enrollment; allocation ratio 1:1:1	Dashboard/chat	Daily	10.2	Overall proportion of participant days that step goals were achieved
	Charity incentives	Upon enrollment; allocation ratio 1:1:1	Dashboard/chat	Daily	10.3	Overall proportion of participant days that step goals were achieved
	Control (no incentives)	Upon enrollment; allocation ratio 1:1:	N/A	N/A	N/A	Overall proportion of participant days that step goals were achieved

^a^1.2=problem solving, 1.4=action planning, 1.6=discrepancy between current behavior and goal, 2.2=feedback on behavior, 4.1=instruction on how to perform a behavior, 10.2=material reward (behavior), and 10.3=nonspecific reward.

^b^N/A: not applicable

**Table 3:** Summary of collected sensor data.

Sensor	Variable	Data type	Frequency^a^
GPS^b^	Location	3D Float	Every 10 min
Accelerometer	Physical activity	Categorical	Continuous
Time	Time	Integer	Continuous
Proximity	Proximity of the phone	Binary (near and far)	Continuous
Wi-Fi	Wi-Fi connection	Categorical/string	Every 10 min
Bluetooth	Bluetooth connection	Categorical/string	Every 10 min
Ambient light	Ambient light	Float	Continuous
Battery status	Battery status	Float (charged in percentage)	Continuous
Screen events	Screen on/off	Binary (on/off)	Continuous

^a^Estimated frequencies only. Actual frequencies may vary depending on device and operating system.

^b^GPS: global positioning system.

For financial and charity incentives, postintervention differences in intrinsic and extrinsic motivation, and differences in app engagement and nonusage attrition during the intervention period are evaluated as additional secondary outcomes. Dimensions of intrinsic and extrinsic motivation are measured using the Behavioral Regulation for Exercise Questionnaire-2 (BREQ-2) [46]. As the external regulation subscale in the BREQ-2 exclusively relates to external regulation by other people, it is substituted by the more generally worded external regulation subscale of the Situational Motivation Scale (SIMS) [47]. Subscales of both instruments have shown good reliability (Cronbach alpha=.73-.86, BREQ-2 [46] and Cronbach alpha=.86, SIMS external regulation subscale [47]). Validity has been confirmed by factor analysis (BREQ-2) [46] and correlational analysis (SIMS) [47]. We measure engagement with the Ally app using the number and length of app launch sessions per day. An app launch session is defined as any interaction of the participant with the Ally app, separated by 5 min between events. If a participant left the app open and did not take action for 5 min or more, then the next interaction with the app counts as a new session. We coded a participant as “non-usage attrition observed” when she/he stopped using the Ally app at least 7 days before the end of the study.

#### Other Outcomes

As a preliminary pre-post evaluation of the Ally app, self-reported health outcomes and targeted mediators of behavior change were assessed at baseline and at postintervention follow-up. In addition, we assessed participant’s perceptions of the Ally app, of intervention components, and of the chatbot in addition to predictors of technology acceptance at postintervention follow-up. An overview of all measured variables is available in Multimedia Appendix 1 ([49-57]).

#### Sensor Data

Drawing on previous literature on context-aware mobile notification management systems [58], we identified smartphone sensors that may aid with predicting the participants’ state of receptivity. Sensor data were obtained from participants during the intervention period. Table 3 provides a summary of these sensors, their collected data, and their sensing frequency. In line with previous studies, we operationalize state of receptivity by using the response rate (ie, whether a participant responds to a notification or not) and the response time (ie, time between notification and response) to notifications of the Ally app.

### Sample Size

**Figure 2:**
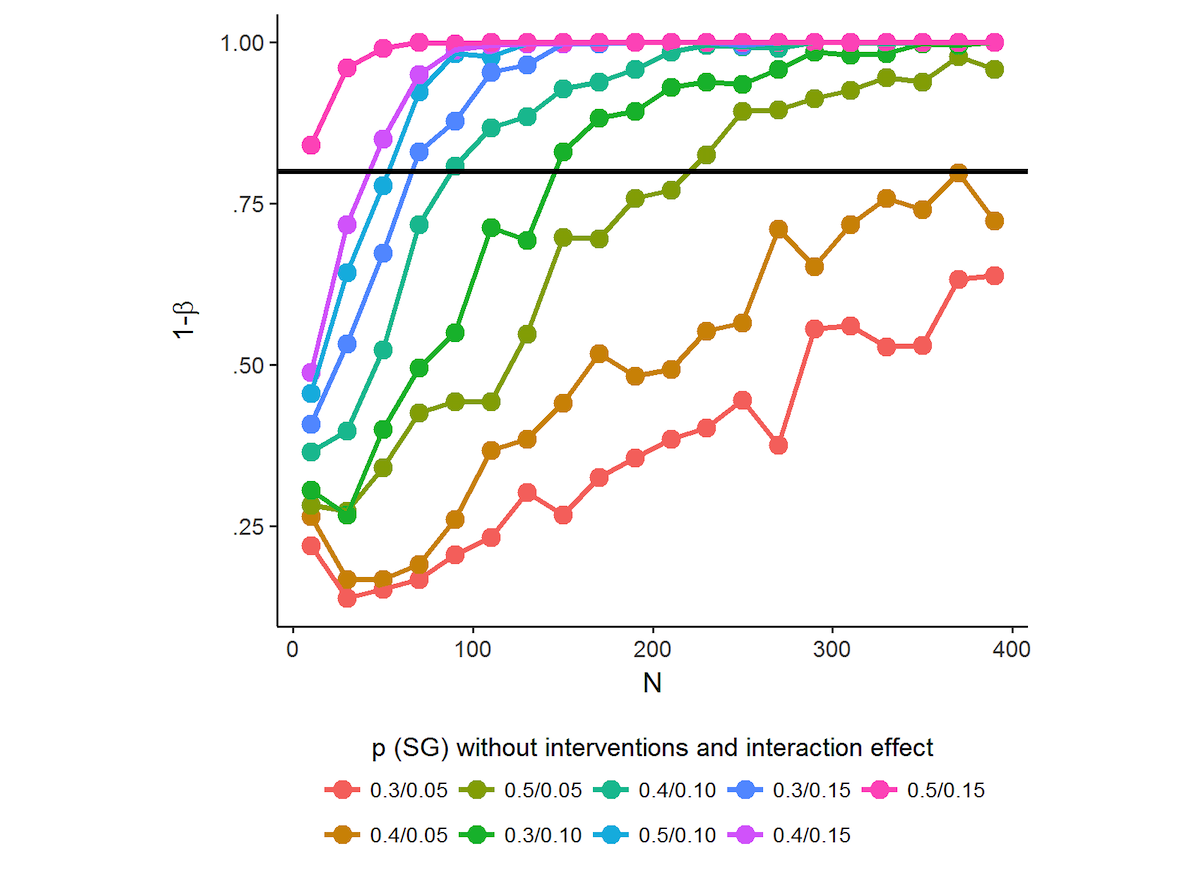
Results of the simulation-based power analysis. p (SG): probability of reaching the step goal.

We used a simulation-based approach to estimate the power of our study design and determine the necessary sample size. As interaction effects require a greater number of participants to be detected with adequate power [59], we focused the power analysis on the two-way interaction effect of the between-subject factor incentives and the within-subject factor planning. We systematically varied the probability of reaching the step goal p (SG) when no intervention is provided (0.30, 0.40, and 0.50). These values seem reasonable given the fact the probability of step goal achievement according to the goal setting algorithm is 0.40. We further varied the increase in probability because of incentive and planning main effects (0.05, 0.10, and 0.15) and the interaction effect (0.05, 0.10, and 0.15) for sample sizes ranging from n=20 to n*=* 400. These effect sizes were based on previous studies on the use of incentives to promote physical activity [38,39]. A total of 100 simulations were generated for each scenario. *P* values of interaction effects were obtained by fitting generalized estimating equations (GEEs) models to the simulated data, and power was calculated as the proportion of *P* values below the significance level of alpha=.05. Figure 2 displays simplified results of this simulation with constant main effects of .15 and different values for p (SG) and the interaction effect. The black horizontal line indicates the recommended level of power of 1-beta=.80.

Simulations indicate that a sample size of roughly 220 is sufficient to detect an interaction effect of .05 with a power of 1-beta=.80 and alpha=.05 for p (SG)=.50. Sample sizes to detect an interaction effect .05 considerably increase for smaller values of p (SG) and smaller main effects (not shown). We, therefore, considered a sample size of 220 to be most feasible, and accounting for dropout, we set the target sample size for our study to 300.

### Recruitment and Eligibility

We invited insurees via email to participate in our study. On the basis of a previous study in the same population and with a similar recruiting process [60], we expected a participation rate of approximately 3%. We initially sent the invitation email to 10,000 insurees. However, because initial participation was lower than expected, an additional 20,000 insurees were invited to meet the required sample size.

The invitation email contained brief information about the study, eligibility criteria, and emphasized the benefits of participation. No details about the different intervention conditions were disclosed to the insurees. By following a link in the invitation mail, interested insurees were forwarded to an online survey platform, where they were screened for eligibility. Eligibility criteria were as follows: (1) German-speaking, (2) aged 18 years or older, (3) enrolled in a complementary insurance program, (4) being free of any medical condition that prohibits increased levels of physical activity, (5) not actively using an activity tracker or a comparable smartphone app, and (6) not working night shifts.

As meeting the first 3 eligibility criteria could be determined from the insurance company’s database, only insurees meeting these criteria were invited to participate. Due to legal regulations in Switzerland, the Ally app could be offered to insurees with complementary health insurance plans only. Note, however, that in Switzerland, 75% of people are enrolled in complementary insurance plans [61]. We excluded insurees working night shifts because interventions were sent out on prespecified times during the day only. Eligible insurees could subsequently obtain detailed information about the goals and study procedures, provide consent to participate, and enroll in the study. After enrollment, participants completed the first online questionnaire and subsequently received a 6-digit code, together with instructions on how to download and install the Ally app. Participants had to enter the code once upon first opening the Ally app to connect survey data and app data and to ensure that only study participants were using the app.

### Statistical Analyses

All analyses were prespecified before enrolling participants into the study. After completion of the study but before starting data analyses, the statistical methods for analyzing the effects of intervention components were changed from hierarchical linear modeling to a GEE-based approach to avoid biased effect estimates [62].

#### Primary Analyses

To evaluate main effects and interactions of intervention components, we will use the centered and weighted GEE approach described in the study by Boruvka et al [62]. This approach guarantees unbiased effect estimates when treatment and moderator variables are time-varying. Statistical models will evaluate each main effect and interaction of intervention components of interest on the components appropriate proximal outcome. For all main effects and interactions that include comparisons of multiple conditions, the main comparisons of interest are between the respective intervention and control conditions.

Missing data on covariates and on the dependent variable will be imputed using multiple imputation, provided the missing at random assumption is justified. We will perform sensitivity analyses to assess the robustness of the results of the primary analyses. These analyses include a per-protocol analysis and an adjusted analysis in which effect estimates are adjusted for a linear trend of time, baseline step count, and covariates of physical activity. For all tests, we use 2-sided *P* values with alpha<.05 level of significance.

#### Secondary Analyses

Secondary analyses focus on the analysis of intervention components on participants’ step counts and on the effects of incentives on app engagement, nonusage attrition, and motivation. Steps per day are analyzed using the same modeling approach as described above. Again, if missing data can be assumed to be missing at random, we plan to impute missing step counts using multiple imputations. As evidence suggests that participant days with less than 1000 steps are unlikely to represent accurate activity data [63,64], those days will be set to missing before imputation.

Generalized linear models will be used to analyze the effect of incentives on engagement and nonusage attrition. One-way analysis of variance (ANOVA) is performed for each subscale of the BREQ-2 to analyze the effect of incentives on the different forms of intrinsic and extrinsic motivation. *P* values will be adjusted according to the Holm-Bonferroni method [65]. If the omnibus test of the ANOVA is significant, we will investigate contrasts between the 3 incentive groups. Again, the main comparison of interest is between the intervention groups and the control group. An overview of all planned statistical analysis is available in Multimedia Appendix 2.

#### Moderators

Due to the lack of existing research in this field, the moderation analyses of main effects are exploratory and may investigate various moderators of intervention components, different forms of operationalizing these moderators, or varying types of relationships (eg, linear or quadratic). Moderations of main effects are investigated by adding a term for the interaction between the main effect and the respective moderator to the statistical model.

#### State of Receptivity

We will compare several different methodological approaches to predict the participants’ state of receptivity. First, we plan to evaluate the performance of supervised learning algorithms in predicting response rate and response time. These algorithms have produced predictions of acceptable accuracy in previous studies on interruptibility [58]. Second, we plan to frame the problem at hand as a classification problem. A classifier will be trained to learn to differentiate between contexts in which the notification is sent (and are assumed to represent nonreceptive contexts) and contexts in which the participant interacts with the app (and in turn are assumed to represent receptive contexts). To this end, we aim to use generalized linear models as a starting point before exploring online learning algorithms that can learn and adapt to each participant’s preferences, and any change thereof. This analysis strategy, however, is preliminary at the time of writing, as the final analysis will consider additional factors such as the quality and distribution of collected data.

## Results

### Recruitment

**Figure 3:**
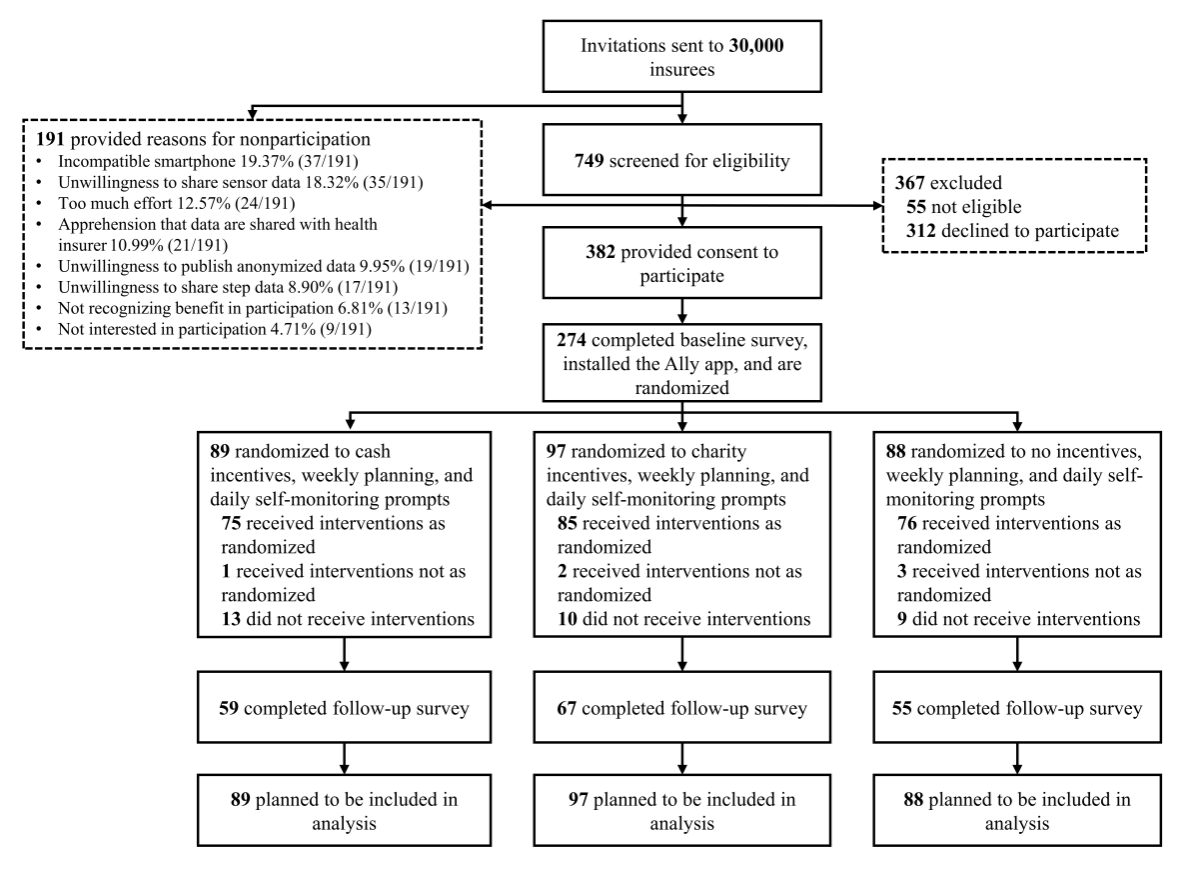
Participant flow.

Of all 30,000 invited insurees, 749 (2.50%) clicked the link in the invitation mail and were subsequently screened for eligibility. Of those, 694 (92.7%) were eligible and 382 (51.0%) provided informed consent to participate. Of all insurees who provided informed consent, 274 (71.7%) successfully completed the baseline survey and installed the Ally app on their smartphone (Figure 3). Invited insurees were given the opportunity to select reasons why they declined participation from a list of predefined answer options using a separate survey (n=191). A link to this survey was included in the invitation mail and placed on the informed consent screen. Possession of an incompatible smartphone (37/191, 19.4%) and unwillingness to share smartphone sensor data (35/191, 18.3%) were the most frequently stated reasons to decline participation.

Of 274 participants, 32 (11.7%) did not receive any interventions because they stopped using the app before the start of the intervention period. Due to technical errors, 6 participants did not receive the interventions they were randomized to (eg, a self-monitoring prompt was sent out on a day where the participant was randomized to not receiving a prompt). For the 6 participants, these errors affected between 1 and 25 out of 42 participant days. Steps per day measured with the smartphone are available for 227 (82.8%, 227/274) participants, and smartphone sensor data are available for 247 (90.1%, 247/274) participants. After completing the 6-week intervention period, 181 (66.1%, 181/274) participants filled out the Web-based follow-up survey. Data collection finished in January 2018.

### Baseline Characteristics

Baseline and demographic characteristics of participants are presented in Table 4. Participants (mean age 41.73 years; 57.7% [158/274] female) were mostly Swiss (246/274, 89.8%) and walked on average 6336 (SD 2701) steps per day during the baseline period. The distribution of age and gender is comparable with those of other studies evaluating physical activity apps [66,67]. Self-reported physical activity and comparisons of self-reported health with the German 12-item Short Form norm sample indicate that on average, participants in our study may be healthier and more active than the general population.

### Expected Results and Dissemination

We will start data analyses after publication of this study protocol. We anticipate submitting results to a peer-reviewed journal in 2019. Preliminary results of the study may be presented at conferences, workshops, symposia, etc. Results of the analysis of sensor data to predict the participants’ state of receptivity will be published separately in a peer-reviewed journal or conference proceedings.

**Table 4:** Baseline and demographic characteristics of participants (N=274).

Characteristics		Statistics
Age in years, mean (SD)		41.73 (13.54)
**Sex, n (%)**		
	Female	158 (57.7)
	Male	111 (40.5)
	N/A^a^	5 (1.8)
**Education, n (%)**		
	Compulsory education	3 (1.1)
	High school	97 (35.4)
	University	164 (59.9)
	N/A	10 (3.7)
**Nationality, n (%)**		
	Swiss	246 (89.8)
	German	13 (4.7)
	Other	12 (4.4)
	N/A	3 (1.1)
**Employment, n (%)**		
	Full-time	152 (55.5)
	Part-time	76 (27.7)
	Retired	22 (8.0)
	Unable to work	2 (0.7)
	Unemployed	14 (5.1)
	N/A	8 (2.9)
**Income, n (%)**		
	<CHF 2500	30 (11.0)
	CHF 2501-5000	53 (19.3)
	CHF 5001-7500	86 (31.4)
	CHF 7501-10,000	37 (13.5)
	>CHF 10,000	24 (8.8)
**Smartphone, n (%)**		
	iPhone operating system	186 (67.9)
	Android	88 (32.1)
**Step count, n (%)**		
	<5000	74 (27.0)
	5000-7499	68 (24.8)
	7500-9999	35 (12.8)
	>10,000	21 (7.7)
	N/A	76 (27.7)
**IPAQ^**b,**^** **, n (%)**		
	Low	31 (11.3)
	Moderate	115 (42.0)
	High	122 (44.5)
	N/A	6 (2.2)
BMI^c^, mean (SD)		24.44 (4.15)
SF-12^d^ physical component summary, mean (SD)		53.32 (4.58)
SF-12 mental component summary, mean (SD)		51.17 (8.11)

^a^N/A: not applicable.

^b^IPAQ: International Physical Activity Questionnaire (short form) [68].

^c^BMI: body mass index.

^d^SF-12: 12-item Short Form.

## Discussion

### Summary

This study protocol describes the design of an MRT that investigates the effectiveness of 3 intervention components as well as associated moderators to guide the design of a smartphone app to promote physical activity. This study is among the first to generate data for the evidence-based development of a JITAI for physical activity. In addition, a data collection strategy is described that enables the parallel collection of sensor data needed to build predictive models that, when implemented into a JITAI, allow real-time prediction of the state of receptivity. These predictions allow to better inform adaptive intervention delivery by highlighting situations where users are likely to respond to intervention notifications. Insights from this study are of value for anyone involved in the development of mHealth interventions and to support important decisions, such as which components to include in an mHealth intervention or how to tailor intervention delivery to participants over time.

### Strengths and Limitations

Our study illustrates potential and challenges associated with mHealth studies. The study’s remote recruitment and data collection process allowed recruiting more than 270 participants in less than a week and collecting a unique and powerful high-resolution dataset that contains real-world behavioral and contextual sensor data. In line with other mHealth studies [69], we observed a larger drop in app usage at the beginning of the study, potentially complicating interpretation of our findings. Likewise, step and sensor data were missing for some participants. Explanations for missing data include never reacting to a message of the Ally chatbot, which was required to request step counts from GoogleFit or the HealthKit, or denying app permissions to collect sensor data. Even though the Ally app instructed participants to carry their smartphone whenever possible, other studies observed an underestimation of smartphone-based step counts because smartphones are often not carried consistently in free-living conditions [70]. This may lead to conservative effect estimates, if increases in step counts are not recorded by the Ally app. Sending invitations via email and to insurees of one insurer only, the restricted range of compatible smartphones, and the requirement to share sensitive data (eg, global positioning system sensor data) are likely contributing to a self-selection of participants in our study. This limits the generalizability of our findings and conclusions. Although all participants indicated upon enrollment that they were using no comparable app or device for tracking physical activity, we cannot exclude that such apps or devices were used or that participants primarily used the Apple Health or GoogleFit apps that were required for the Ally app to count steps correctly. Use of such additional apps or devices could potentially affect the use of the Ally app and the effectiveness of intervention components.

If intervention components prove to be effective, we plan to include them in a revised version of the Ally app that provides just-in-time adaptive support depending on identified moderators and predicted states of receptivity. We plan to evaluate this revised version in a randomized controlled trial.

## Appendix

Multimedia Appendix 1 Study timeline including intervention components and assessment of outcomes.

Multimedia Appendix 2 Overview of variables, measures and methods of analysis.

## Abbreviations

Ally: Assistant to Lift your Level of activitY
ANOVA: analysis of variance
AP: action planning
BREQ-2: Behavioral Regulation for Exercise Questionnaire-2
CP: coping planning
CC: control condition (no planning)
GEE: generalized estimating equation
JITAI: just-in-time adaptive intervention
mHealth: mobile health
MRT: microrandomized trial
p (SG): probability of reaching the step goal
SIMS: Situational Motivation Scale
