# Supplementary material for: Investigating Intervention Components and Exploring States of Receptivity for a Smartphone App to Promote Physical Activity: Protocol of a Microrandomized Trial
Source: JMIR Res Protoc. 2019 Jan 31;8(1):e11540. doi: 10.2196/11540 (PMC6374735; doi:10.2196/11540)
Supplement: Multimedia Appendix 1 [file resprot_v8i1e11540_app1.pdf]

Study timeline including intervention components and assessment of outcomes

	Assessment instrument	Measurement	Timepoint				
			Enrolment	Allocation	Baseline	Intervention period	Post-intervention follow-up
**Enrolment**							
Eligibility screen	-	-	X				
Informed consent	-	-	X				
Allocation	-	-		X			
**App components**							
Dashboard	-	-			X	X	
Step goals	-	-				X	
Self-monitoring prompts	-	-				X	
Planning	-	-				X	
Incentives	-	-				X	
**Primary outcome**							
Goal achievement	Smartphone	Step goal; steps per day				X	
**Secondary outcomes**							
Steps	Smartphone	Steps per day			X	X	
App engagement	Smartphone	Number and length of app launch sessions per day				X	
Non-usage attrition	Smartphone	App usage stopped until 7 days before end of study				X	
Motivation	Self-report	Behavioral Regulation in Exercise Questionnaire-2 (BREQ-2 [46]), Situational Motivation Scale (SIMS [47])			X		X
**Other outcomes**							
Physical and mental health status	Self-report	12-Item Short Form Health Survey (SF-12 [49])			X		X
Physical activity	Self-report	International Physical Activity Questionnaire – short form (IPAQ (short form) [68])			X		X
Body mass index	Self-report	Height; weight			X		X
Stage of change	Self-report	Adopted from Lippke et al. [50]			X		X
Planning	Self-report	Adopted from Sniehotta et al. [29]			X		X
Task self-efficacy	Self-report	Adopted from Scholz et al. [51]			X		X
Maintenance self-efficacy	Self-report	Adopted from Schwarzer et al. [52]			X		X
Action control	Self-report	Adopted from Sniehotta et al. [24]			X		X
Adoption intention	Self-report	Adopted from Venkatesh et al. [53]			X		
Continued use intention	Self-report	Adopted from Venkatesh et al. [54]					X
Performance expectancy	Self-report	Single item based on the scale in Venkatesh et al. [54]					X

	Assessment instrument	Measurement	Timepoint				
			Enrolment	Allocation	Baseline	Intervention period	Post-intervention follow-up
Effort expectancy	Self-report	Single item based on the scale in Venkatesh et al. [54]					X
Hedonic motivation	Self-report	Single item based on the scale in Venkatesh et al. [54]					X
Habit	Self-report	Single item based on the scale in Venkatesh et al. [54]					X
Overall satisfaction	Self-report	Single item					X
Evaluation of intervention components	Self-report	Single items					X
Positive aspects of the app	Self-report	Open-ended question					X
Negative aspects of the app	Self-report	Open-ended question					X
Improvement suggestions	Self-report	Open-ended question					X
Appropriateness of message frequency	Self-report	Single item					X
Attitudes towards the Ally chatbot	Self-report	Adopted from Bickmore et al. [55]					X
**Participant characteristics**							
Age	Self-report	Single item			X		
Gender	Self-report	Single item			X		
Education	Self-report	Single item			X		
Employment	Self-report	Single item			X		
Income	Self-report	Single item			X		
Nationality	Self-report	Single item			X		
Neighborhood characteristics	Self-report	European environmental questionnaire (ALPHA short [56])			X		
Big 5 personality traits	Self-report	10-item Big Five Inventory (BFI-10 [57])			X		
**Sensor data**							
GPS	Smartphone	-				X	
Accelerometer	Smartphone	-				X	
Proximity	Smartphone	-				X	
Wi-Fi	Smartphone	-				X	
Bluetooth	Smartphone	-				X	
Ambient light	Smartphone	-				X	
Battery status	Smartphone	-				X	
Screen events	Smartphone	-				X	
