# Supplementary material for: Investigating Intervention Components and Exploring States of Receptivity for a Smartphone App to Promote Physical Activity: Protocol of a Microrandomized Trial
Source: JMIR Res Protoc. 2019 Jan 31;8(1):e11540. doi: 10.2196/11540 (PMC6374735; doi:10.2196/11540)
Supplement: Multimedia Appendix 2 [file resprot_v8i1e11540_app2.pdf]

Overview of variables, measures and methods of analysis

Variable/outcome	Hypothesis	Outcome measure	Method of analysis
1) Primary outcome			
Participant days that step goals are achieved	Financial incentives increase the total proportion of participant days that step goals are achieved	Step goal achievement (binary)	Weighted and centered GEE
	Charity incentives increase the total proportion of participant days that step goals are achieved		
	Action planning increases the weekly proportion of participant days that step goals are achieved		
	Coping planning increases the weekly proportion of participant days that step goals are achieved		
	Self-monitoring prompts increase the daily proportion of participant days that step goals are achieved		
	The effect of action planning on goal achievement is increased for participants receiving incentives and vice versa		
	The effect of coping planning on goal achievement is increased for participants receiving incentives and vice versa		
	The effect of self-monitoring prompts on goal achievement is increased for participants receiving financial incentives and vice versa		
	The effect of self-monitoring prompts on goal achievement is increased for participants receiving charity incentives and vice versa		
2) Secondary outcomes			
Steps	Cf. hypotheses for primary outcome	Steps per day (continuous)	Weighted and centered GEE
Engagement	Incentives increase engagement	Number of app launch sessions (continuous);<br>Length of app launch sessions (continuous)	GLM
Non-usage attrition	Incentives decrease non-usage attrition	Indicator whether participant stopped using Ally until 7 days before the end of the study (binary)	GLM
Intrinsic regulation	Incentives affect intrinsic motivation	Behavioural Regulation in Exercise Questionnaire-2 (BREQ-2)	ANOVA
Identified regulation	Incentives affect identified regulation	Behavioural Regulation in Exercise Questionnaire-2 (BREQ-2)	ANOVA
Introjected regulation	Incentives affect introjected regulation	Behavioural Regulation in Exercise Questionnaire-2 (BREQ-2)	ANOVA
External regulation	Incentives affect external regulation	Situational Motivation Scale (SIMS)	ANOVA
3) Sensitivity analyses			
Per protocol analysis			
Adjusting for covariates			
